# miR-590-3 and SP1 Promote Neuronal Apoptosis in Patients with Alzheimer's Disease via AMPK Signaling Pathway

**DOI:** 10.1155/2021/6010362

**Published:** 2021-12-15

**Authors:** Yanqun Cao, Xiangxiang Tan, Quzhe Lu, Kai Huang, Xiaoer Tang, Zhiming He

**Affiliations:** ^1^Shaoyang University Basic Medical College, Shaoyang 422000, Hunan Province, China; ^2^Shaoyang University School of Nursing, Shaoyang 422000, Hunan Province, China

## Abstract

Alzheimer's disease (AD) is a progressive neurological degenerative illness with a hidden onset. Its pathogenesis is complicated, although with molecular biology research on cancer and targeted research on pathogenic mechanisms, good progress has not yet been made. Therefore, this work built a multifactor-driven neuronal apoptosis dysfunction module for the purpose of probing its underlying pathogenic mechanisms. We performed differential expression analysis, coexpression analysis, enrichment analysis, and hypergeometric tests to calculate the underlying regulatory effects of multifactors on the modules by the way of the whole gene expression profile of AD and identify a series of ncRNA (miR-320a) and TF (NFKB1). Additionally, we screened 10 modules corresponding to the Hub gene, which tend to regulate the physiological progress of inflammation, regulation of autophagy, cerebral cortex neuron differentiation, glial cell apoptotic, and so on. Meanwhile, Alzheimer's disease is triggered by signaling pathways such as the MPK signaling pathway. In this study, a dysfunction module is utilized to verify that miR-590-3 and SP1 motility factors can regulate neurons in Alzheimer's disease through the MPK signaling pathway, not only providing new insights into the pathogenesis of Alzheimer's disease but also laying a solid theoretical foundation for the biologists to further cure Alzheimer's disease.

## 1. Introduction

Alzheimer's disease (AD), a chronic neurodegenerative disease, usually occurs at a slow rate and deteriorates with time [1]. So far, there have been no effective drugs to cure or delay it [2]. Therefore, it is urgent for research on the pathogenesis and treatment mechanism of Alzheimer's disease. Fortunately, many biologists and medical researchers have devoted themselves to the exploration of the pathogenesis, physiological processes, and treatments of Alzheimer's disease, which made great achievements. For example, Choi et al. found that glucocorticoids are the leading risk factor for Alzheimer's disease, severely causing microtubule instability and cognitive impairment, which are considered as an early pathological feature that ultimately results in memory deficits [3]. Besides, there is increasing evidence that the pathogenesis of Alzheimer's disease is intricate and involves various biological mechanisms, such as amyloid-based neurodegeneration that affects degenerative diseases [4, 5]. Therefore, it is effective for targeting these proteins to prevent neurodegeneration and protect nerves [6]. In previous studies, it was found that the formation of neurofibrillary tangles (NFTs) is the central mark of AD, and an NFT is a twisted fiber that takes shape in brain cells. Some factors regulate insulin signaling pathways to involve the pathogenesis of AD, GSK 3*β*, JNK, CaMKII, CDK 5, CK1, Mark4, PLK 2, Syk, DYRK1A, PPP, p70S6K, and other mechanisms contributing to the formation of neurotoxic A*β* and NFT in the brain [7]. It is worth noting that synaptic plasticity and cognitive decline are the most outstanding characteristics of Alzheimer's disease [8]. In brief, synaptic loss is an early pathological manifestation, and cognitive decline is currently the best correlative factor with Alzheimer's disease [9]. Recently, evidence suggests that neuronal inflammation stimulated by microglia (macrophage-like immune cells in the brain) serves a vital function in the pathogenesis of chronic encephalitis, and the fact that neuroinflammation is likely to be one of the key factors causing Alzheimer's disease is reliably proved by the postmortem brain tissue in AD patients [10]. Furthermore, sphingosine-1-phosphate (S1P) is a pleiotropic bioactive lipid regulating many pathophysiological processes, inflammation included. Spinster homolog 2 (Spns2), an S1P transporter, facilitates the proinflammatory activation of microglia in vitro and in vivo, contributing to accelerating the pathogenesis of Alzheimer's disease [11]. Salazar et al.'s study indicates that the risk factor PyK 2 (PTK2B) is particularly positioned on neurons in the adult brain and that the Pyk 2 risk gene is directly associated with neuronal amyloid-*β*-oligomer (A*β*o) signaling pathways to impair synaptic anatomy and function. Therefore, Pyk 2 serves a central function in AD-related synaptic dysfunction by mediating A*β*-triggered dysfunction [12].

With the molecular studies of Alzheimer's disease, some key genes have been discovered as therapeutic targets. Through the report of Tian et al., we know that oxidative stress is related to the pathogenesis of Alzheimer's disease. When oxidative stress becomes more and more obvious, the endogenous protective pathway of nuclear factor E2 related factor 2 (Nrf2)/antioxidant response element (ARE) was reduced in PS1V97L-Tg mice of 10 months old [13]. In the original astrocytes and brain lysates of AD patients, KCa3.1 expression was significantly connected with endoplasmic reticulum (ER) stress and unfolded protein response (UPR). KCa3.1 also regulated Ca2+ homeostasis in astrocytes and weakened UPR as well as ER stress, resulting in memory deficits and neuronal loss [14]. On the other hand, biologists have also identified a series of Alzheimer's disease-related signaling pathways affecting all kinds of physiological processes and mediating the occurrence of diseases. As described in the studies by Cisternas et al., Wnt signaling disorders are connected with dominating neurodegenerative diseases, and the fact that neuroprotective effects of Wnt signaling in AD mouse models are at least partially improved by Wnt-mediated neuronal glucose metabolism is known from the animal experiments [15]. Moreover, L-3-n-butylphthalide (L-NBP) can inhibit neuronal apoptosis, which serves a neurogenic function in various animal and cell models. PI3K/Akt may be a target in the process by upregulating cyclin D1 to encourage neural stem cell proliferation [16]. A study found that JAK/STAT 3 signaling pathways mediate the development and progression of neuroinflammation in the neurons of patients with Alzheimer's disease [17]. Furthermore, the AMPK/SIRT 1 signaling pathway is regulated by dihydromyricetin (DHM), which serves as a protection in AD by upregulating the AMPK/SIRT1 pathway, inhibiting inflammatory responses and hippocampal cell apoptosis, and improving cognitive function [18]. These new gene targets offer a new therapeutic approach to Alzheimer's disease. However, further exploration is still required for a comprehensive understanding of the basic detailed mechanisms and key molecular targets of Alzheimer's disease.

Here, we combine data of patients with Alzheimer's disease and normal people to further explore the underlying pathogenesis of Alzheimer's disease and finally conclude that miR-590-3 and SP1 are key regulatory genes to Alzheimer's disease, which can encourage neuronal apoptosis in patients with Alzheimer's disease through the AMPK signaling pathway. Therefore, the comprehensive strategy based on functional modules not only helps to explore the potential pathogenic molecular mechanisms of Alzheimer's disease but also offers rich resources and theoretical guidance for biologists to further explore its therapeutic mechanisms.

## 2. Materials and Methods

### 2.1. Related Gene Expression Profile of Alzheimer's Disease

The data of Alzheimer's disease-related expression profile was obtained from the NCBI Gene Expression Omnibus (GEO) database [19]. Among them, GSE85426 contains 90 cases of healthy people and 90 cases of patients with Alzheimer's disease. Besides, the basic data processing package of the R language expression profiling chip (including R.utils, R.oo, R.methodsS3, and hgu133plus2cdf) were applied to construct disease and normal sample expression profiles for 180 samples, calculated by the R language limma package [20]. For chip data, primarily background correction and standardization were performed by the background correct function. Then, the control probe and the low-expressed probe are filtered using the quantile normalization method based on the normalized between arrays function to obtain high quality of standardized data. The lmFit and eBayes functions of the limma package were analyzed with default parameters to identify differentially expressed genes with *p* value > 0.01, and finally 433 differential genes were obtained.

### 2.2. Coexpression Analysis

For exploring the synergistic expression of these human apoptosis-related genes in Alzheimer's disease neurons, we employed weighted gene coexpression network analysis (WGCNA) [21] to investigate the gene expression profile of human apoptosis-related genes and look for gene modules for synergistic expression. Unlike general clustering methods, it is biologically significant for the WGCNA clustering criteria. Accordingly, the correlation coefficient of the intergene expression level is taken as *n* power so that the distribution of the correlation coefficient values gradually conforms to the scale-free distribution. Then, on the basis of cohesion, a hierarchical clustering tree is built by calculating correlation coefficients among genes. Genes with similar patterns are grouped into the same branch, and various branches of the cluster tree stand for various gene modules. Therefore, the results obtained by this method are more credible. In this study, 11 coexpression modules were obtained with the gray modules removed to obtain the Hub gene corresponding to 10 modules.

### 2.3. Analysis of Functional and Pathway Enrichment

Exploring the function and signaling pathways involving genes is often a practical means of studying the molecular mechanisms of disease. Hence, the enrichments of Go function (pvalueCutoff = 0.01, qvalueCutoff = 0.01) and the KEGG pathway (pvalueCutoff = 0.05, qvalueCutoff = 0.2) were performed for the 10 modules of the gene using the R language clusterProfiler package. Moreover, we apply the Cytoscape application to conduct functional analysis for the integrated module network.

### 2.4. Identification of the Regulation of ncRNA and TF on Modules

For exploring the driving force of the coexpression module of neuronal apoptosis-related genes in Alzheimer's disease, we employed the ncRNA-miRNA (protein) interaction pairs with a score ≥ 0.5 in the RAID v2.0 database and downloaded from the TRRUST v2.0 database. All data of human transcription factor target were utilized as background sets for pivotal analysis. Pivot analysis refers to finding a driver with at least two interactions corresponding to the module in a target pair and calculating the significance of the interaction between the driver and the module according to the hypergeometric test. The ncRNA with *p* value < 0.01 was screened for the pivot of the significant regulatory module. Finally, statistical analysis of pivots was conducted. Pivots that regulated more dysfunctional modules were authentically identified as core pivots. The data based on ncRNA and TF targets were predicted as background sets, and the pivotal regulator of the regulatory dysfunction module was obtained.

## 3. Result

### 3.1. Analysis of Gene Differential Expression of Alzheimer's Disease

Gene expression dysregulation features in the process of disease. For the purpose of exploring the genetic disorder of Alzheimer's disease, we screened the expression profiles of Alzheimer's disease and obtained 433 differentially expressed genes (*p* < 0.01). These differentially expressed genes may be directly or indirectly in touch with Alzheimer's disease, which is probably important in the development of the disease.

### 3.2. Coexpression Behavior of Related Genes of Human Apoptosis in Alzheimer's Disease

**Figure 1:**
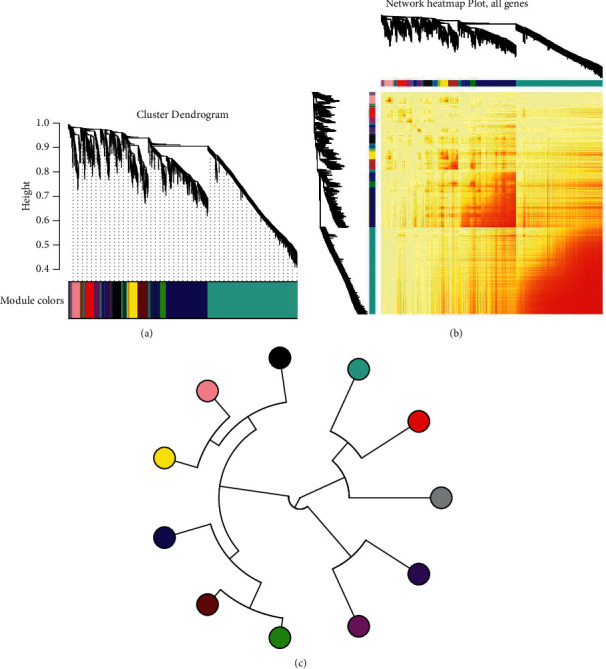
(a) Coexpression analysis clusters related genes of human apoptosis into 11 modules, with 11 colors standing for different modules. (b) Cluster expression heat map of the module genes in the sample. (c) Cluster analysis tree between modules, with different colors representing different modules.

For the purpose of systematically studying the mechanism of action of Alzheimer's disease-related genes in patients' samples, we conducted massive analytical studies. Primarily, we built expression profiles with 4571 human apoptosis-related genes in patient samples. Then, on the basis of analysis of coexpression network, we obtained expression states of neuronal apoptosis-related modules in 11 groups of Alzheimer's disease patients (Figures 1(a)–1(c)). These gene sets were authentically as 11 dysfunction modules. Besides, these 11 modules were screened, and the gray module was removed. Eventually, the hub genes corresponding to 10 modules were captured. These dysfunction modules may engage in various functions and pathways on behalf of the situation of various regulatory mechanisms mediating neurons of Alzheimer's disease.

### 3.3. Identification of Pathogenic Modules Based on Function and Pathway

**Figure 2:**
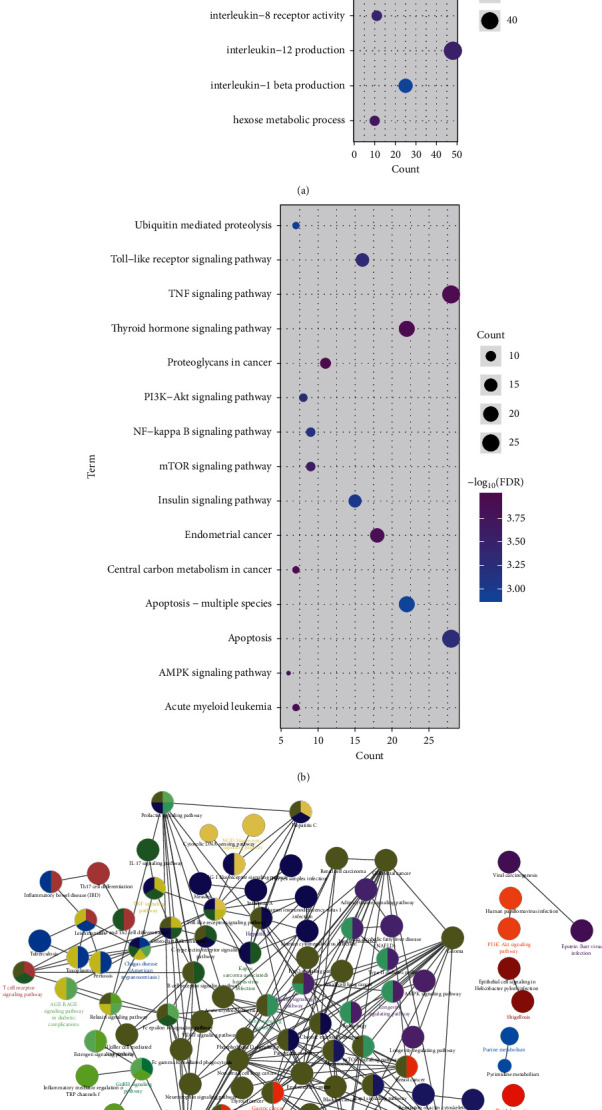
Functions and pathways involving modular genes identify neuronal apoptosis and dysfunction modules of Alzheimer's disease. Analysis excerpt of GO function enrichment in module genes. The deeper the colors, the stronger the enrichment. The larger the circle, the greater the proportion of the module genes account for the entry gene of GO function. (a, b) Analysis excerpt of the KEGG pathway enrichment in module genes. The deeper the colors, the stronger the enrichment. The larger the circle, the greater the proportion of the module genes account for the entry gene of the KEGG pathway. (c) The corresponding functional and access networks according to the relationships between the modules were utilized to identify the proportion of the corresponding functions and pathways involving modules.

It is an important medium for identifying their pathogenesis to study the functions and pathways involved with genes. In order to study the possible dysfunction of the modular gene imbalance, we performed analysis of GO function and KEGG pathway enrichment for 11 modules. We gathered abundant GO terms, totaling 5061 cell composition entries, 8280 molecular functional terms, as well as 48822 biological processes (Figure 2(a)). On the basis of the functional analysis, we observed that relevant functional modules have a tendency to enrich multiple disease-related functions. For example, regulation of autophagy, cerebral cortex neuron differentiation, and glial cell apoptotic process. On the other hand, the 48822 KEGG pathway enrichment results (Figure 2(b)) illustrated that the functional module genes are chiefly engaged in the AMPK signaling pathway, apoptosis, the PI3K-Akt signaling pathway, and the NF-kappa B signaling pathway. These signaling pathways have been proved to be inextricably linked to neurons of Alzheimer's disease. Since the functional and pathway results obtained by enrichment of the module genes are closely connected with the apoptosis of neurons in Alzheimer's disease, we determined these 11 modules as dysfunction modules. Module genes can regulate a range of functions and pathways, and module dysregulation is likely to be an important inducement of morbidity. Looking back at the whole effect of these modules, we constructed a functional network of all modules in conjunction with the relationships between the modules (Figure 2(c)), which may stand for a global dysfunctional mechanism for neurons of Alzheimer's disease. The dysregulation of genes within the module triggers dysfunction of the module, which in turn influences the functions and pathways involved, inducing the occurrence and progression of the disease.

### 3.4. ncRNA That Mediates Dysfunction Modules

**Figure 3:**
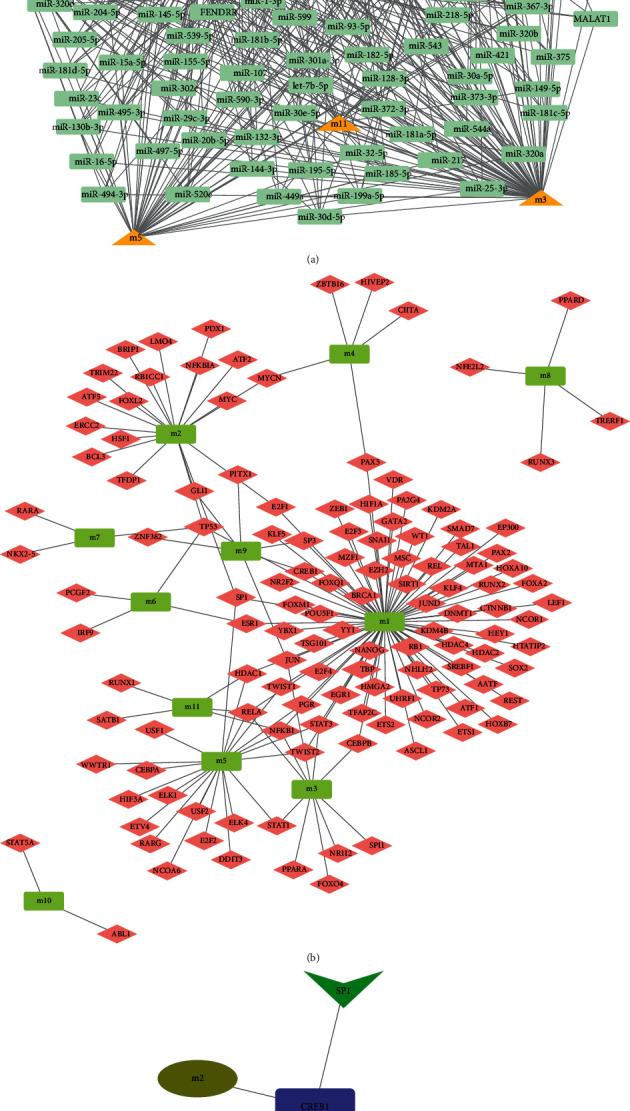
Related dysfunction module in the neuronal apoptosis of Alzheimer's disease that pivot regulator mediates. (a) Network diagram of the adjustment of ncRNA to neuronal apoptosis-related modules in Alzheimer's disease. (b) Network diagram of transcription factors regulating related functional modules of neuronal apoptosis in Alzheimer's disease. (c) Identification of key genes of Alzheimer's disease may affect neuronal apoptosis in patients with Alzheimer's disease through corresponding signaling pathways.

The transcription and post-transcriptional regulation of genes have long been regarded as key factors to regulate the occurrence and development of diseases, and ncRNA is thought to be an important regulator. Scientific prediction of ncRNAs that regulate dysfunction module genes facilitates our further investigation of the transcriptional regulation mechanisms of neurons in Alzheimer's disease. Thus, kindly I scatter, the pivot analysis of pRNA was employed to seek for the ncRNA regulators that cause dysfunction of the module. The predicted results (Schedule 2, Figure 3(a)) manifested that 2027 ncRNAs have a dramatic regulatory impact on the module, referring to1327 ncRNA-Module target pairs. These ncRNAs affect the apoptosis of neurons in Alzheimer's disease to varying degrees. Besides, statistical analysis of the results found that miR-320a have distinguished regulatory functions on 9 dysfunctional modules, vital in the dysfunction of the module. However, miR-148b-3p, miR-182-5p, and miR-200c-3p have been identified and have important regulatory roles in eight dysfunction modules, which may become potential apoptotic factors in neurons in patients with Alzheimer's disease. Other than that, ncRNAs also exhibit distinguished modulation for dysfunction modules and have an effect essentially on the apoptosis of neurons in patients with Alzheimer's disease.

### 3.5. Identification of Key Target Genes Based on TF-Pivot

The apoptosis in patients with Alzheimer's disease is also inseparable from the dysregulation of transcription factors, which is also embodied in the regulation of transcription factors on dysfunction modules. Therefore, we performed a pivot analysis of the module on the basis of the regulatory relationship of the transcription factor to the gene. The results illustrate that (Schedule 3, Figure 3(b)), a total of 128 transcription factors have distinguished transcriptional regulation for dysfunction modules of neuronal apoptosis in Alzheimer's disease, referring to 157 TF-Module regulatory pairs. Statistical analysis of the regulatory roles of these transcription factors reveals that HIF1A, NFKB1, RELA, and TP53 prominently regulate 4 dysfunction modules, which may join in the inflammatory process and accelerateneuronal apoptosis in patients with Alzheimer's disease. However, JUN, HDAC1, SP1, and STAT3 are also indispensable in the neuronal apoptosis mechanism of patients with Alzheimer's disease. Reviewing the global (Figure 3(c)), we also found that the key genes miR-590-3 and SP1 affecting neurons in Alzheimer's disease may jointly contribute to the apoptosis of neurons in Alzheimer's disease by targeting CREB1 to mediate the involvement of module 2 in the AMPK signaling pathway and enhance the neuronal apoptosis in patients with Alzheimer's disease.

## 4. Discussion

Alzheimer's disease (AD), a progressive neurodegenerative disease, features cognitive decline and dementia [22, 23]. In recent years, the exploration of Alzheimer's disease has focused on certain genes or proteins, as well as related signaling pathways, and certain achievements have been made. However, the global regulation of these genes, proteins, and signaling pathways in Alzheimer's disease remains unclear. For the purpose of comprehensively exploring the mechanism of action of potential pathogenic genes in Alzheimer's disease, primarily these potential pathogenic genes as well as their interaction genes were integrated and observed for their coordinated expression behavior in disease patient samples. Thus, we obtained 11 coexpression modules. Meanwhile, these 11 modules were screened, and the gray module was removed. Eventually, 10 modules corresponding to 10 Hub genes were captured. It has been found that the Hub gene GSTP1 of the sixth module is considered to be a risk factor for Alzheimer's disease. This view is also sided by studies of scientists such as Wang M. thatGSTP1 polymorphism may raise the risk of Alzheimer's disease [24]. Further, we also observed that the Hub gene adiponectin receptor 1 (ADIPOR1) of block 7 as a permeation protein receptor may be associated with the Nogo-A and Nogo-66 receptor 1 (NgR1) to develop a complex that inhibits long-term potentiation and cognitive function. Osmotic proteins are able to encourage neurite outgrowth as well as synaptic complexity through AdipoR1 and NgR1 signaling, suggesting that AdipoR1 may be an available therapeutic target for neurodegenerative diseases like Alzheimer's disease [25].

Next, on the basis of the results of enrichment analysis, we found that genes of two modules (RELA and TLR4) are chiefly engaged in the regulation of autophagy. Among them, Zahmatkesh and other scholars have reported that autophagy stress is linked to Alzheimer's disease, which is also considered to be a potential cause of nerve loss [26]. Therefore, the regulation of autophagy features in the pathogenesis of Alzheimer's disease. In another aspect, the enrichment result of the pathway reveals that the functional module gene chiefly engages in the neuronal apoptosis triggered by the AMPK signaling pathway in Alzheimer's disease. Studies have demonstrated that TRPML1 may regulate autophagy to engage in the pathogenesis of AD through the AMPK signaling pathway [27]. Additionally, Wang et al. confirmed that the type 2 cannabinoid receptor (CB2R) joins in AD pathology in a mouse model, and it turns out that AD-like tau protein is hyperphosphorylated. In the meantime, neurodegenerative lesions including phosphorylated tau protein are also manifested in biologists such as Tapia-Rojas C. After being verified, deletion of CB2R has induced behavioral impairment and AD-like pathology alternation via the AMPK pathway [28, 29]. Therefore, it is proved that the functions and pathways involving these module genes produce a comprehensive network effect, comprehensively regulating the pathogenesis of Alzheimer's disease.

We then explored substantial drivers for these dysfunction modules. Transcription factors based on NFKB1, RELA, and TP53 are important regulatory factors for dysfunction modules. First, inflammation is the main mechanism of acute brain injury and chronic neurodegeneration, whereas neuroinflammation is basically regulated by the transcription factor NF-*κ*B, which is closely connected with the occurrence of Alzheimer's disease [30]. Secondly, the pathology of AD is often accompanied by an inflammatory response, and RELA (P65), an important signaling factor in the NF-*κ*B pathway, is usually involved in the regulation of neuroinflammation to mediate the development and progress of Alzheimer's disease [31]. Then, the tumor suppressor TP53 is a key protein of neurodegenerative diseases and cancer, which can downregulate specific autophagy-related mitotic responses through transcriptional inhibition of PINK1 [32]. Therefore, these studies have proved that these driving factors are important for regulating the occurrence and development of Alzheimer's disease. Moreover, in terms of the driving force of ncRNA, we predicted that multiple miRNAs such as miR-320a, miR-182-5p, and miR-200c-3p serve as important mediators for dysfunction modules. Studies by Denk et al. have confirmed that miR-320a is also associated with the protein biomarker amyloid *β*1-42 and phosphorylated neurofilament heavy chain levels, suggesting its potential effect in progressive neuronal degeneration monitoring [33]. The difference of synaptic plasticity is one of the earliest expressions of neurodegenerative states clinically. Many of the microRNAs (miRNAs) are enriched in synapses, possibly regulating local protein synthesis in a rapid response to stressors like replicating prions, which may be a candidate modulator of neurodegenerative changes. Boese et al. found an increase in miRNAs that are dysregulated in the later stages of the disease by mimicking infected animals (AD model), including misalignment of miR-182-5p and miR-200c-3p [34].

## 5. Conclusion

In our analysis process, we also found that the drivers of Alzheimer (miR-590-3 and SP1) may target the module genes and have an important regulatory effect. Thus, we boldly speculated that its involvement in the regulation of transforming growth factor-*β*1 (TGF-*β*1) is decreased in the hippocampus of AD mice, causing impairment of memory function and neuronal apoptosis in Alzheimer's disease [35].

In light of the results of this study, we obtained a more comprehensive dysfunction module for neuronal apoptosis in Alzheimer's disease. These modules provided a number of genes proved to be linked with Alzheimer's disease and candidate factors to be tested, supplying a theoretical basis for further research on Alzheimer's disease. After systematic research, we believe that miR-590-3 and SP1 are key regulatory genes for Alzheimer's disease, which can promote neuronal apoptosis in patients with Alzheimer's disease through the AMPK signaling pathway.

## Data Availability

The datasets used and/or analyzed during the current study are available from the corresponding author on reasonable request.

## References

[1] Chen F. Z., Zhao Y., Chen H. Z. (2019). MicroRNA-98 reduces amyloid beta-protein production and improves oxidative stress and mitochondrial dysfunction through the Notch signaling pathway via HEY2 in Alzheimer’s disease mice. *International Journal of Molecular Medicine*.

[2] Yao Y., Wang Y., Kong L., Chen Y., Yang J. (2019). Osthole decreases tau protein phosphorylation via PI3K/AKT/GSK-3beta signaling pathway in Alzheimer’s disease. *Life Sciences*.

[3] Choi G. E., Oh J. Y., Lee H. J. (2018). Glucocorticoid-mediated ER-mitochondria contacts reduce AMPA receptor and mitochondria trafficking into cell terminus via microtubule destabilization. *Cell Death & Disease*.

[4] Griffin E. F., Yan X., Caldwell K. A., Caldwell G. A. (2018). Distinct functional roles of Vps41-mediated neuroprotection in Alzheimer’s and Parkinson’s disease models of neurodegeneration. *Human Molecular Genetics*.

[5] Galvão F., Grokoski K. C., da Silva B. B., Lamers M. L., Siqueira I. R. (2019). The amyloid precursor protein (APP) processing as a biological link between Alzheimer’s disease and cancer. *Ageing Research Reviews*.

[6] Kianpour Rad S., Arya A., Karimian H. (2018). Mechanism involved in insulin resistance via accumulation of beta-amyloid and neurofibrillary tangles: link between type 2 diabetes and Alzheimer’s disease. *Drug Design, Development and Therapy*.

[7] Hu Y., Chen W., Wu L. (2018). TGF-beta1 restores hippocampal synaptic plasticity and memory in alzheimer model via the PI3K/Akt/Wnt/beta-Catenin signaling pathway. *Journal of Molecular Neuroscience: M*.

[8] Luchena C., Zuazo-Ibarra J., Alberdi E., Matute C., Capetillo-Zarate E. (2018). Contribution of neurons and glial cells to complement-mediated synapse removal during development, aging and in Alzheimer’s disease. *Mediators of Inflammation*.

[9] Thawkar B. S., Kaur G. (2019). Inhibitors of NF-kappaB and P2X7/NLRP3/Caspase 1 pathway in microglia: novel therapeutic opportunities in neuroinflammation induced early-stage Alzheimer’s disease. *Journal of Neuroimmunology*.

[10] Zhong L., Jiang X., Zhu Z. (2018). Lipid transporter Spns2 promotes microglia pro-inflammatory activation in response to amyloid-beta peptide. *Glia*.

[11] Salazar S. V., Cox T. O., Lee S. (2018). Alzheimer’s disease risk factor Pyk2 mediates amyloid-beta induced synaptic dysfunction and loss. *The Official Journal of the Society for Neuroscience*.

[12] Tian Y., Wang W., Xu L. (2018). Activation of Nrf2/ARE pathway alleviates the cognitive deficits in PS1V97L-Tg mouse model of Alzheimer’s disease through modulation of oxidative stress. *Journal of Neuroscience Research*.

[13] Yu Z., Dou F., Wang Y., Hou L., Chen H. (2018). Ca(2+)-dependent endoplasmic reticulum stress correlation with astrogliosis involves upregulation of KCa3.1 and inhibition of AKT/mTOR signaling. *Journal of Neuroinflammation*.

[14] Cisternas P., Zolezzi J. M., Martinez M., Torres V. I., Wong G. W., Inestrosa N. C. (2018). Wnt-induced activation of glucose metabolism mediates the in vivo neuroprotective roles of Wnt signaling in Alzheimer disease. *Journal of Neurochemistry*.

[15] Wang S., Huang L., Zhang Y., Peng Y., Wang X., Peng Y. (2018). Protective effects of L-3-n-butylphthalide against H2O2-induced injury in neural stem cells by activation of PI3K/akt and Mash1 pathway. *Neuroscience*.

[16] An X.-q., Xi W., Gu C.-y., Huang X. (2018). Complement protein C5a enhances the beta-amyloid-induced neuro-inflammatory response in microglia in Alzheimer’s disease. *Médecine/Sciences*.

[17] Sun P., Yin J. B., Liu L. H. (2019). Protective role of Dihydromyricetin in Alzheimer’s disease rat model associated with activating AMPK/SIRT1 signaling pathway. *Bioscience Reports*.

[18] Aminyavari S., Zahmatkesh M., Farahmandfar M., Khodagholi F., Dargahi L., Zarrindast M.-R. (2019). Protective role of Apelin-13 on amyloid beta25-35-induced memory deficit; Involvement of autophagy and apoptosis process. *Progress in Neuro-Psychopharmacology and Biological Psychiatry*.

[19] Yu G., Wang L.-G., Han Y., He Q.-Y. (2012). clusterProfiler: an R package for comparing biological themes among gene clusters. *OMICS: A Journal of Integrative Biology*.

[20] Han H., Cho J.-W., Lee S. (2018). TRRUST v2: an expanded reference database of human and mouse transcriptional regulatory interactions. *Nucleic Acids Research*.

[21] Yi Y., Zhao Y., Li C. (2017). RAID v2.0: an updated resource of RNA-associated interactions across organisms. *Nucleic Acids Research*.

[22] Flores J., Noël A., Foveau B., Lynham J., Lecrux C., LeBlanc A. C. (2018). Caspase-1 inhibition alleviates cognitive impairment and neuropathology in an Alzheimer’s disease mouse model. *Nature Communications*.

[23] Wang M., Li Y., Lin L., Song G., Deng T. (2016). GSTM1 null genotype and GSTP1 Ile105Val polymorphism are associated with Alzheimer’s disease: a meta-analysis. *Molecular Neurobiology*.

[24] Yoon G., Shah S. A., Ali T., Kim M. O. (2018). The adiponectin homolog osmotin enhances neurite outgrowth and synaptic complexity via AdipoR1/NgR1 signaling in Alzheimer’s disease. *Molecular Neurobiology*.

[25] Zhang L., Fang Y., Cheng X. (2017). TRPML1 participates in the progression of Alzheimer’s disease by regulating the PPARgamma/AMPK/mtor signalling pathway. *Cellular Physiology and Biochemistry*.

[26] Wang L., Liu B.-J., Cao Y. (2018). Deletion of type-2 cannabinoid receptor induces Alzheimer’s disease-like tau pathology and memory impairment through AMPK/GSK3beta pathway. *Molecular Neurobiology*.

[27] Vargas J. Y., Fuenzalida M., Inestrosa N. C. (2014). In vivo activation of Wnt signaling pathway enhances cognitive function of adult mice and reverses cognitive deficits in an Alzheimer’s disease model. *Journal of Neuroscience*.

[28] Rolova T., Puli L., Magga J. (2014). Complex regulation of acute and chronic neuroinflammatory responses in mouse models deficient for nuclear factor kappa B p50 subunit. *Neurobiology of Disease*.

[29] Xie L., Jiang C., Wang Z. (2016). Effect of Huperzine A on Abeta-induced p65 of astrocyte in vitro. *Bioscience Biotechnology and Biochemistry*.

[30] Checler F., Goiran T., Alves da Costa C. (2018). Nuclear TP53: an unraveled function as transcriptional repressor of PINK1. *Autophagy*.

[31] Denk J., Oberhauser F., Kornhuber J. (2018). Specific serum and CSF microRNA profiles distinguish sporadic behavioural variant of frontotemporal dementia compared with Alzheimer patients and cognitively healthy controls. *PLoS One*.

[32] Boese A. S., Saba R., Campbell K. (2016). MicroRNA abundance is altered in synaptoneurosomes during prion disease. *Molecular and Cellular Neuroscience*.

[33] Barrett T., Wilhite S. E., Ledoux P. (2013). NCBI GEO: archive for functional genomics data sets--update. *Nucleic Acids Research*.

[34] Ritchie M. E., Phipson B., Wu D. (2015). Limma powers differential expression analyses for RNA-sequencing and microarray studies. *Nucleic Acids Research*.

[35] Langfelder P., Horvath S. (2008). WGCNA: an R package for weighted correlation network analysis. *BMC Bioinformatics*.

